# Association of COVID-19 and Arterial Stiffness Assessed using Cardiovascular Index (CAVI)

**DOI:** 10.2174/0115734021279173240110095037

**Published:** 2024-03-27

**Authors:** Valery Podzolkov, Anna Bragina, Aida Tarzimanova, Lyubov Vasilyeva, Ilya Shvedov, Natalya Druzhinina, Yulia Rodionova, Tatiana Ishina, Iuliia Akyol, Valentina Maximova, Alexandr Cherepanov

**Affiliations:** 1 Department of Faculty Therapy No. 2, Sechenov First Moscow State Medical University (Sechenov University), Moscow, Russia

**Keywords:** Arterial stiffness, cardiovascular index, COVID-19, cardiovascular risk factors, hypertension, diabetes mellitus

## Abstract

**Background:**

COVID-19 is characterized by an acute inflammatory response with the formation of endothelial dysfunction and may affect arterial stiffness. Studies of cardio-ankle vascular index in COVID-19 patients with considered cardiovascular risk factors have not been conducted.

**Objective:**

The purpose of our study was to assess the association between cardio-ankle vascular index and COVID-19 in hospitalized patients adjusted for known cardiovascular risk factors.

**Methods:**

A cross-sectional study included 174 people hospitalized with a diagnosis of moderate COVID-19 and 94 people without COVID-19. Significant differences in the cardio-ankle vascular index values measured by VaSera VS - 1500N between the two groups were analyzed using parametric (Student's t-criterion) and nonparametric (Mann-Whitney) criteria. Independent association between COVID-19 and an increased cardio-ankle vascular index ≥ 9.0 adjusted for known cardiovascular risk factors was assessed by multivariate logistic regression.

**Results:**

There were significantly higher values of the right cardio-ankle vascular index 8.10 [7.00;9.40] and the left cardio-ankle vascular index 8.10 [6.95;9.65] in patients undergoing inpatient treatment for COVID-19 than in the control group – 7.55 [6.60;8.60] and 7.60 [6.60;8.70], respectively. A multivariate logistic regression model adjusted for age, hypertension, plasma glucose level, glomerular filtration rate and diabetes mellitus showed a significant association between increased cardio-ankle vascular index and COVID-19 (OR 2.41 [CI 1.09;5.30]).

**Conclusion:**

Hospitalized patients with COVID-19 had significantly higher cardio-ankle vascular index values compared to the control group. An association between an increased cardio-ankle vascular index and COVID-19 was revealed, independent of age, hypertension, plasma glucose level, glomerular filtration rate and diabetes mellitus.

## 1 INTRODUCTION

Coronaviridae is a family of viruses whose members are responsible for outbreaks of severe acute respiratory syndrome (SARS) in 2002-2004, Middle East respiratory syndrome (MERS) in 2012 and the novel coronavirus disease COVID-19 (COronaVIrus Disease 2019) [1-3]. The latter caused a pandemic with over 765 million confirmed cases and over 6.9 million deaths. Despite the fact that the World Health Organisation (WHO) had declared the end of COVID-19 as a global health emergency, there were over 1.2 million new cases and over 7,100 deaths in the last months [4-6]. High mortality is noted in patients with a history of cardiovascular diseases, including arterial hypertension (HTN) [7-10]. COVID-19 is characterized by an acute inflammatory response with the release of a wide range of cytokines, risk of a cytokine storm, and endothelial damage [11, 12]. The virus, with its own spike-glycoproteins, binds Angiotensin-converting enzyme type 2 (ACE2) receptor on the surface of endotheliocytes and enters the cells [13-15]. The development of endotheliitis and endothelial cell apoptosis involving matrix metalloproteinases was described earlier [16-18]. This results in the decreased expression of ACE2 and reduced conversion of Angiotensin II to Angiotensin 1-7, promoting further imbalance in the Renin-Angiotensin-Aldosterone and Kallikrein-Kinin systems [19, 20]. It contributes to the formation of endothelial dysfunction with increased adhesion of leukocytes, activation of the complement system, increased vascular permeability, platelet aggregation, hypercoagulation, and synthesis and secretion of pro-inflammatory cytokines [21-23]. Systemic inflammation causes an imbalance of hormones and vascular resistance [24, 25]. This leads to the hyporeactivity of vascular adrenergic receptors, a decrease in the level of endogenous vasopressin, and a lack of corticosteroids [26]. As a result, COVID-19 may alter arterial stiffness [15, 27-30]. Earlier studies have shown a significantly higher arterial stiffness index and pulse wave velocity (PWV) in COVID-19 non-survivors compared to survivors and the association between increased pulse pressure and in-hospital mortality [31-34]. Studies have revealed a high prevalence of cardiovascular diseases and risk factors in COVID-19 patients [35-38]. Arterial stiffness is an important predictor of cardiovascular events and all-cause mortality [39-42]. Age, HTN, and other cardiovascular factors are associated with increased arterial stiffness, which, in combination with systemic inflammation, can worsen the course of COVID-19 [28, 31, 32].

On the other hand, increased COVID-19-related arterial stiffness can worsen the prognosis for post-COVID patients [43, 44]. According to the results of several studies, a large number of patients (from 35% to 90.5%) develop a post-COVID condition [45, 46]. Although the most frequent post-COVID-19 symptoms are fatigue, dyspnea, neuropsychological disorders, and pain, patients present altered glucose levels, blood pressure and lipid profiles several months after recovery from COVID-19 infection [47, 48]. Some studies showed cardiovascular events were more frequent in post-COVID patients, so people older than eighteen years are at an increased risk of developing new-onset incidents of diabetes, hypertension, or dyslipidaemia several months after COVID-19 infection [47-52]. Arterial stiffness is a predictor of cardiovascular diseases; it can be considered a marker of post-COVID syndrome. One of the indicators of arterial stiffness is the cardio-ankle vascular index (CAVI), which was developed in 2004. It is relatively simple and inexpensive to measure [53]. According to the American Heart Association Statement and the Consensus of Russian experts, CAVI can be used for the evaluation of arterial stiffness along with pulse wave velocity [54, 55]. A number of studies assessed and presented age reference values of CAVI in a healthy population [56-58]. Experts highlight the clinical value of CAVI as a predictor of hypertension, cardiovascular diseases, kidney injury progression, cognitive decline and dementia, and cardiovascular and total mortality [59]. Furthermore, there is data that CAVI may be superior in predicting all-cause mortality, major cardiovascular outcomes and renal function decline compared to PWV [60]. The study of the validity of atherosclerotic calcified lesions demonstrated that CAVI can be used to assess atherosclerotic changes at all stages of disease progression [61]. Studies of CAVI in COVID-19 patients with considered cardiovascular risk factors have not been conducted. The purpose of our study was to assess the association between CAVI and COVID-19 in hospitalized patients adjusted for known cardiovascular risk factors.

## 2 MATERIALS AND METHODS

### 2.1 Study Design and Patients

A cross-sectional study was conducted at the COVID hospital and the therapeutic department of the University Clinical Hospital Nº4 of Sechenov University in accordance with the Declaration of Helsinki on Human Rights and the approval of the Local Ethics Committee (protocol number 01-22). All participants signed an informed consent prior to any procedure included in the study protocol. The study cohort comprised 268 people over the age of 18 years. The case group included 174 people hospitalized with a diagnosis of moderate COVID-19. The control group included 94 people without COVID-19 and with no signs of an infectious disease, who were undergoing examination and treatment in the therapeutic department. COVID-19 was diagnosed using a positive polymerase chain reaction (PCR) test. Exclusion criteria were age under 18 years, pregnancy, peripheral arterial disease and other known arterial diseases, chronic kidney disease with glomerular filtration rate (GFR) less than 30 ml/min, malignancy, a permanent form of atrial fibrillation, coronary heart disease, the use of invasive mechanical ventilation. In all participants, we recorded age, sex, smoking, comorbidities, and concomitant medications. The evaluation was conducted in a quiet room in the sitting position and repeated three times with a 2-minute gap after 10 minutes of rest. Fasting blood samples for evaluation of leukocytes, glucose, and creatinine levels were obtained from the median vein of the elbow. GFR was calculated using the Chronic Kidney Disease Epidemiology Collaboration equation (CKD-EPI).

### 2.2 Group Characteristics

The characteristics of the cohort are presented in Table ****1****. There were no significant differences in age and gender distribution between patients with COVID-19 and controls, so the two groups were comparable in these parameters. More than half of patients with COVID-19 (62.43%) had hypertension. Median blood pressure in this group was systolic – 120.00 [120.00; 130.00] and diastolic – 77.00 [70.00;80.00] mm Hg. More than a half (55.17%) of the case group were receiving antihypertensive therapy: 43.10% were taking Renin-Angiotensin-Aldosterone System (RAAS) inhibitors, 6.02%-monotherapy with beta-blockers, 0.60% - monotherapy with calcium channel blockers, 34.94%-combined antihypertensive therapy. The rate of hypertension (75.53%), systolic blood pressure (137.50 [125.00; 150.00]) and diastolic blood pressure (80.00 [80.00; 90.00]) were significantly higher in the control group *vs* COVID-19 patients. 46.81% of patients without COVID-19 were taking RAAS inhibitors, 3.33% - monotherapy with beta-blockers, 0.00% - monotherapy with calcium channel blockers, 45.56% - combined antihypertensive therapy. Оverweight and obese patients were prevalent in both groups. Body mass index did not significantly differ between patients with COVID-19 and non-COVID patients. Thus, the groups were comparable in this parameter. There were no significant differences in the rate of diabetes mellitus between groups, the plasma glucose level was higher in the case group. In the COVID-19 group, 94.71% of patients were treated with dexamethasone, 39.66% of patients – with inhibitors of cytokine signaling (Tocilizumab, Levilimab, *etc*.), 67.82% of patients required oxygen therapy, 1.72% of patients were admitted to an intensive care unit, none required intubation during hospitalization. The degree of lung injury was assessed by a chest computed tomography (CT) scan. It was classified as CT-0 (mild) to CT-4 (critical) according to the Consensus Guidelines of the Russian Society of Radiology (RSR) and the Russian Association of Specialists in Ultrasound Diagnostics in Medicine (RASUDM) [62]. There were 51 (29.31%) patients with a mild degree, 100 (57.47%) patients with a moderate degree, 20 (11.49%) patients with a severe degree and 3 (1.72%) patients with a critical degree of lung damage in COVID-19 group. The rate of smokers was higher in the control group. Blood leucocyte count and glomerular filtration rate did not statistically differ between groups. The duration of COVID-19 infection was 6.00 [4.00; 9.^50^] days.

### 2.3 Evaluation of Arterial Stiffness

The parameters of arterial stiffness were measured by sphygmography using the VaSera VS - 1500N (Fukuda Denshi, Japan). All subjects were examined in the morning, having abstained from alcohol, caffeine, and smoking for 8 hours prior to the measurement. The procedure was conducted in a supine position, registering cuff-based four-limb PWV and recording both a phonocardiogram and the lead II of an electrocardiogram simultaneously. The metrics of arterial stiffness, *i.e.*, CAVI in the right (R-CAVI) and left (L-CAVI) extremities, were calculated; arithmetic mean CAVI values were calculated as well. A CAVI value of ≥ 9.0 was considered increased [53].

### 2.4 Statistical Analysis

Statistical analysis was performed using Statistica 12.0 (StatSoft Inc). All continuous variables were tested for the normal distribution using the Kolmogorov-Smirnov test and were presented as means with a standard deviation or medians with interquartile ranges (IQRs). Categorical variables are expressed as absolute and relative frequencies. Statistically significant differences in continuous variables were analyzed using parametric (Student's t-criterion) and nonparametric (Mann-Whitney) criteria. To compare the categorical variables of two independent groups, the χ^2^ test was used. The correlation between arterial stiffness values, COVID-19, and other parameters was assessed in all study participants using the Pearson correlation coefficient. A multivariate logistic regression was used to estimate the independent association of an increased CAVI ≥ 9.0 with COVID-19 adjusted for known cardiovascular risk factors. Odds ratios (ORs) with 95% confidence intervals (CI) were calculated to estimate the strength of the relationship. A *p*-value < 0.05 was considered statistically significant.

## 3 RESULTS

There were significantly higher values of R-CAVI (8.10 [7.00; 9.^40^] *vs.* 7.55 [6.60; 8.^60^], *p* = 0.011) and L-CAVI (8.10 [6.95; 9.^65^] *vs.* 7.60 [6.60; 8.^70^], *p* = 0.009) in patients undergoing inpatient treatment for COVID-19 than in the control group (Table **1**).

We assessed the potential predictors of a high CAVI by correlation analysis. Correlation analysis demonstrated significant correlations of average CAVI values with COVID-19 (r = 0.162, *p* = 0.008), age (r = 0.661, *p* <0.001), diabetes mellitus (r = 0.140, *p* = 0.023), systolic blood pressure (r = 0.151, *p* = 0.015), hypertension (r = 0.343, *p*<0.001), plasma glucose level (r = 0.211, *p* <0.001), GFR (CKD-EPI) (r = -0.423, *p*<0.001), correlations with sex (r = 0.017, *p* = 0.781), body mass index (r = -0.044, *p* = 0.475), smoking (r = 0.024, *p* = 0.735), heart rate (r = 0.037, *p* = 0.548), leucocytes (r = -0.002, *p* = 0.977) were insignificant. Also, we revealed a significant correlation of average CAVI values with plasma glucose level (r = 0.174, *p* <0.023) in the COVID-19 group.

A multivariate logistic regression model was used to study the association between the increased CAVI and COVID-19 adjusted for other confounders detected by correlation analyses, OR per unit increase with corresponding 95% CI was used as a measure of association. Significant associations of increased CAVI have been identified with age – OR 1.10 [1.06; 1.^15^] and COVID-19 – OR 2.83 [1.12; 7.^10^]. Associations with systolic blood pressure - OR 1.01 [0.99; 1.0^3^], hypertension – OR 1.31 [0.53; 3.^25^], plasma glucose level – 1.08 [0.91; 1.^28^], GFR (CKD-EPI) – 0.99 [0.96; 1.0^2^], and diabetes mellitus – 1.30 [0.47; 3.^61^] were non-significant (Table **2**).

## 4 DISCUSSION

Arterial stiffness is a potent cardiovascular predictor that increases the risk of such cardiovascular events as death, stroke, myocardial infarction, and heart failure [63]. COVID-19, both in acute phase and post-acute sequelae is often accompanied by cardiometabolic risk factors: hypertension, overweight, obesity, fasting hyperglycemia, and diabetes mellitus [64-66]. All these conditions are associated with increased arterial stiffness [67, 68]. Thus, the interaction between the course of COVID-19 and arterial stiffness may interfere with the prognosis of COVID-19 survivors.

We found a significantly higher CAVI in the group of hospitalized COVID-19 patients compared to the control group. That result is independent of age, hypertension and diabetes mellitus, plasma glucose levels, and GFR. It should be emphasized that both groups were comparable in age and major cardiometabolic risk factors. Moreover, there was a slightly higher rate of hypertension and smoking in the control group. Although smoking could potentially contribute to greater arterial stiffness, the level of CAVI was greater in the COVID-19 group.

Arterial stiffness in COVID-19 has been assessed in some studies, but measured by other methods and in other populations. For instance, Szeghy R. *et al*. (2021) revealed a significantly higher aortic augmentation index in young patients with COVID-19, determined by the method of applanation tonometry, compared with young healthy volunteers. The study enrolled only 15 people without chronic diseases or cardiovascular risk factors [69]. Similar findings of significantly higher PWV in COVID-19 patients compared to healthy young volunteers were identified in a small study by Ratchford S. *et al.* (2021) (11 and 20 patients, respectively) [70]. A comparison of indicators reflecting arterial stiffness in patients with COVID-19 in combination with diabetes mellitus and hypertension has been carried out in a number of studies. In their papers, Schnaubelt S. (2021) and Faria D. (2023) revealed significantly higher rates of PWV in patients with COVID-19 in relation to the control group of comparable age, sex, and comorbidities. The studies involved small cohorts of 22 and 19 people in each group, respectively [71, 72]. A larger cohort of patients was analyzed in the study by Stamatelopoulos K. *et al.* (2021). The authors collected data from three large studies, calculated PWV based on mean blood pressure and age data, and obtained a new sampling of 233 pairs of COVID-19-positive patients and the control group, comparable in terms of cardiovascular risk factors. As a result, it was demonstrated that the estimated PWV values in COVID-19 patients were significantly higher than in patients without COVID-19 [73].

The reference standard for the evaluation of arterial stiffness is PWV measurement [54, 63, 73]. An alternative method for the estimation of arterial stiffness is the calculation of CAVI [55, 74]. The measurement of CAVI has several advantages. First of all, it is independent of blood pressure. Secondly, the ascending aorta is represented in the measurement. Thirdly, the technique is relatively simple, less operator-dependent, fast, and inexpensive to use, which is essential for COVID-19 testing and treatment [53, 75]. For this reason, the assessment of arterial stiffness by determining CAVI was highly prioritized in our work.

The level of CAVI in COVID-19 patients has been studied only by Aydin E. *et al*. (2021). Results have shown significantly higher CAVI values in 65 COVID-19 patients compared to the control group of 50 people without COVID-19 [30]. The key differences from our sample were the older age of the study participants and the presence of coronary heart disease. In addition, that work did not take into account the fact of smoking, which is fundamental in the increase of arterial stiffness [76], or assess the strength and independence of the association of the studied factors with arterial stiffness. A multivariate logistic regression with adjustment for these factors showed an independent association between the increased CAVI and COVID-19, which indicates the importance of the coronavirus disease for arterial stiffness formation. A close relationship between arterial stiffness and worsened cardiovascular prognosis is widely documented. The methodology of our study cannot provide the answer to the question of reversibility of the detected high arterial stiffness. However, few prospective studies have shown that increased PWV and augmentation index persist for at least a year after the severe acute respiratory syndrome caused by the coronavirus 2 (SARS-CoV-2) infection [77, 78].

The limitations of our study were its single-center nature, namely, the focus on the population of hospitalized patients with COVID-19, thus excluding less severely affected ambulatory patients and the fact that the control group was smaller than the study group. Also, we excluded patients with several conditions specified in the exclusion criteria, which could possibly impact the selection bias.

## CONCLUSION

Thus, hospitalized patients with moderate COVID-19 had significantly higher CAVI values compared to the control group. An association between an increased CAVI and COVID-19, independent of age, hypertension and diabetes mellitus, plasma glucose levels, and GFR may add to the prognosis of both acute and post-acute COVID-19.

## Figures and Tables

**Table 1:** Characteristics of the cohort.

**-**	**COVID-19 Patients (n=174)**	**Control Group (n=94)**	***p***
Age, years	58.41 ± 12.94	55.18 ± 13.10	0.053
Sex: male/female, n (%)	78 (44.83)/96 (55.17)	32 (34.04)/62 (65.96)	0.087
Smoking, n (%)	17 (9.77)	27 (28.72)	0.007
DM, n (%)	30 (17.34)	11 (11.83)	0.235
HTN, n (%)	108 (62.43)	71 (75.53)	0.030
RAAS blockers, n (%)	75 (43.10)	44 (46.81%)	0.464
BMI, kg/m^2^	28.60 [25.90; 31.60]	30.64 [25.99; 34.65]	0.142
Systolic BP, mm Hg	120.00 [120.00; 130.00]	137.50 [125.00; 150.00]	<0.001
Diastolic BP, mm Hg	77.00 [70.00; 80.00]	80.00 [80.00; 90.00]	<0.001
HR, bpm	82.00 [75.00; 90.00]	74.50 [69.00; 80.00]	<0.001
Leucocytes, *10^9^/L	6.59 [4.20; 10.36]	6.70 [5.70; 7.85]	0.322
Plasma glucose, mmol/L	5.73 [5.00; 6.72]	5.30 [4.70; 5.90]	0.002
GFR (CKD-EPI), ml/min/1,73m^2^	74.65 ± 15.86	71.67 ± 17.68	0.170
C-reactive protein, mg/L	24.50 [10.30; 59.00]	-	-
R-CAVI	8.10 [7.00; 9.40]	7.55 [6.60; 8.60]	0.011
L-CAVI	8.10 [6.95; 9.65]	7.60 [6.60; 8.70]	0.009

**Abbreviations:** DM, diabetes mellitus; HTN, arterial hypertension; RAAS, Renin-Angiotensin-Aldosterone System; BMI, body mass index; BP, blood pressure; HR, heart rate; GFR (CKD-EPI), glomerular filtration rate calculated using the Chronic Kidney Disease Epidemiology Collaboration equation; R-CAVI, cardio-ankle vascular index (right); L-CAVI, cardio-ankle vascular index (left).

**Table 2:** Results of a multiple logistic regression model with respect to the increased CAVI ≥ 9.0.

**-**	**OR**	**CI 95% Lower**	**CI 95% Upper**	***p***
Age	1.10	1.06	1.15	<0.001
Systolic BP	1.01	0.99	1.03	0.312
HTN	1.31	0.53	3.25	0.566
Plasma glucose	1.08	0.91	1.28	0.365
GFR (CKD-EPI)	0.99	0.96	1.02	0.540
DM	1.30	0.47	3.61	0.616
COVID-19	2.83	1.12	7.10	0.027

**Note:** CAVI, cardio-ankle vascular index; HTN, arterial hypertension; GFR (CKD-EPI), glomerular filtration rate calculated using the Chronic Kidney Disease Epidemiology Collaboration equation; DM, diabetes mellitus; OR, odds ratio; CI 95% lower, lower 95% confidence interval; CI 95% upper, upper 95% confidence interval.

## Data Availability

The data supporting the findings of the article are available from the corresponding author upon reasonable request.

## LIST OF ABBREVIATIONS

SARS: Severe Acute Respiratory Syndrome
MERS: Middle East Respiratory Syndrome
ACE2: Angiotensin-Converting Enzyme Type 2
CAVI: Cardio-Ankle Vascular Index
PCR: Polymerase Chain Reaction
GFR: Glomerular Filtration Rate
CKD-EPI: Chronic Kidney Disease Epidemiology Collaboration Equation
RSR: Russian Society of Radiology
RASUDM: Russian Association of Specialists in Ultrasound Diagnostics in Medicine
IQRs: Interquartile Ranges
